# Glycolysis mediates neuron specific histone acetylation in valproic acid-induced human excitatory neuron differentiation

**DOI:** 10.3389/fnmol.2023.1151162

**Published:** 2023-04-06

**Authors:** Andi Chen, Mengmeng Wang, Chao Xu, Youyi Zhao, Panpan Xian, Yuqian Li, Weian Zheng, Xuyang Yi, Shengxi Wu, Yazhou Wang

**Affiliations:** ^1^Department of Neurobiology, School of Basic Medicine, Institute of Neurosciences, Fourth Military Medical University, Xi’an, Shaanxi, China; ^2^State Key Laboratory of Military Stomatology and National Clinical Research Center for Oral Diseases and Shaanxi Engineering Research, Center for Dental Materials and Advanced Manufacture, Department of Anesthesiology, School of Stomatology, Fourth Military Medical University, Xi’an, Shaanxi, China; ^3^School of Life Sciences and Research Center for Natural Peptide Drugs, Shaanxi Engineering and Technological Research Center for Conversation and Utilization of Regional Biological Resources, Yan’an University, Yan’an, China

**Keywords:** valproic acid, human embryonic stem cells, differentiation, glycolysis, histone acetylation

## Abstract

Pregnancy exposure of valproic acid (VPA) is widely adopted as a model of environmental factor induced autism spectrum disorder (ASD). Increase of excitatory/inhibitory synaptic transmission ratio has been proposed as the mechanism of VPA induced ASD. How this happened, particularly at the level of excitatory neuron differentiation in human neural progenitor cells (NPCs) remains largely unclear. Here, we report that VPA exposure remarkably inhibited human NPC proliferation and induced excitatory neuronal differentiation without affecting inhibitory neurons. Following VPA treatment, mitochondrial dysfunction was observed before neuronal differentiation, as showed by ultrastructural changes, respiratory complex activity, mitochondrial membrane potential and oxidation levels. Meanwhile, extracellular acidification assay revealed an elevation of glycolysis by VPA stimulation. Interestingly, inhibiting glycolysis by 2-deoxy-d-glucose-6-phosphate (2-DG) efficiently blocked the excitatory neuronal differentiation of human NPCs induced by VPA. Furthermore, 2-DG treatment significantly compromised the VPA-induced expression of H3ac and H3K9ac, and the VPA-induced binding of H3K9ac on the promoter of *Ngn2* and *Mash1*, two key transcription factors of excitatory neuron fate determination. These data, for the first time, demonstrated that VPA biased excitatory neuron differentiation by glycolysis-mediated histone acetylation of neuron specific transcription factors.

## 1. Introduction

Toxic exposure during early phase of pregnancy usually leads to abnormal neural development. Autism spectrum disorder (ASD) is the most prevalent developmental psychiatric disease which affects approximately 1% of children around the world (Lyall et al., 2017). Exposure to valproic acid (VPA), an anti-epilepsy drug, is widely used as an environmental factor-induced ASD model (Chaliha et al., 2020). Many studies, including ours, have been carried out on VPA-treated mice and proposed that increase of excitatory/inhibitory synaptic transmission may be the major pathological change of VPA-induced ASD (Gogolla et al., 2009; Li et al., 2021; Qi et al., 2022). This disturbance of excitatory/inhibitory ratio could be due to either enhancement of excitatory synaptic function or an overproduction of excitatory neurons. Given that VPA is exposed at the beginning of neural tube formation when neuronal differentiation begins (Kim et al., 2011), it is highly possible that VPA may act at the point of neuron fate determination, which, however, has been poorly investigated.

During development, neuronal cell fate is determined mainly by the expression of key neuron-specific transcription factors (Konstantinides and Desplan, 2020; Pai and Moore, 2021). The expression of neuron-specific transcription factors are coordinated by extracellular neurogenic factors and intra-nuclear epigenetic modifications (Vieira et al., 2018, 2019). Histone acetylation, an important epigenetic modification which closely associates with gene transcription, has been thought to play important roles in neural stem cell differentiation (Coskun et al., 2012; Lilja et al., 2013). As a histone deacetylase inhibitor, VPA reportedly induces neurogenesis of hippocampal and cortical neural stem cells *via* regulating the histone acetylation of neuron-specific transcription factors, such as Ngn1 (Yu et al., 2009; Zhang et al., 2016). Recently, more and more evidence emerged that cytoplasmic metabolic status, namely mitochondrial function, also involves in the process of neuronal differentiation (Khacho et al., 2019). The ratio of glycolysis/oxidative phosphorylation determines the choice of neural progenitors between self-renewal and differentiation (Zheng et al., 2016). Whether mitochondrial function or metabolic status contributed to the effects of VPA on neuronal differentiation remains largely unknown.

In the present study, we addressed this issue by using a well-established human ESC cell line, H8. Our data demonstrated an early differentiation effect of VPA treatment on excitatory neuron differentiation, and surprisingly revealed an involvement of glycolysis in modifying the histone acetylation of excitatory neuron-specific transcription factor Ngn2 and Mash1.

## 2. Materials and methods

### 2.1. Chemicals, antibodies, culture medium, and kits

The culture medium and chemicals used were as the following: mTeSR1 (Cat. 85850, STEMCELL Technologies, Canada), Accutase (Cat. 07920, STEMCELL Technologies, Canada), STEMdiff SMADi Neural Induction Kit (Cat. 08582, STEMCELL Technologies, Canada), Matrigel (Cat. 354277, Corning, New York, USA), rosette selection reagent (Cat. 05832, STEMCELL Technologies, Canada), Neurobasal Medium (Cat. 21103-049, Gibco, USA), DF12 medium (Cat. D8437, Sigma-Aldrich, St. Louis, MO, USA), B27 (Cat. 17504044, Gibco, USA), BNDF (Cat. 45002, Peprotech, USA), GDNF (Cat. 45010, Peprotech, USA), Valproic acid sodium salt (VPA, Cat. P4543, Sigma-Aldrich, St. Louis, MO, USA), 2-Deoxy-D-glucose (2-DG, Cat. PC-43587, PlantChemMed Biology Co., Ltd., Shanghai, China).

The antibodies used were as the following: rabbit anti-Sox2 (Cat. GTX101507, 1:100, GeneTex, USA), rabbit anti-Pax6 (Cat. GTX113241, 1:100, GeneTex, USA), rabbit anti-Ki67 (Cat. GTX16667, 1:100, GeneTex, USA), mouse anti-Tuj-1 (Cat. GT11710, 1:100, GeneTex, USA), rabbit anti-Neurogenin 2 antibody (Cat. GTX129258, 1:1000, GeneTex, USA), mouse anti-CaMKII antibody (Cat. ab22609, 1:100, Abcam, Cambridge, UK), rabbit anti-Mash1 (Cat. ab74065,1:100, Abcam, Cambridge, UK), rabbit anti-VGLUT2 (Cat. 71555, 1:100, Cell signaling technology, Danvers, MA, USA), rabbit anti-Histone-H3 Polyclonal antibody (Cat. 17168-1-AP, 1:3000, Proteintech, Rosemont, IL, USA), donkey anti-rabbit (Alexa Fluor 594, Cat. A-21207, 1:800, Invitrogen, California, USA), donkey anti-mouse (Alexa Fluor 594, Cat. A-21203, 1:800, Invitrogen, California, USA), rabbit anti-Histone-H3ac (Cat. 616378, 1:3000, Active Motif, Carlsbad, CA, USA), mouse anti-Histone-H3K9ac (Cat. 91104, 1:3000, Active Motif, Carlsbad, CA, USA). DAPI (D9542, 1:1000, Sigma-Aldrich, St. Louis, MO, USA), HRP-conjugated anti-rabbit IgG (bs-0296G, 1:5000, Bioss, California, USA), HRP-conjugated anti-mouse IgG (PB002, 1:5000, Zhonghuihecai, Shaanxi, China), ECL kit (Cat. 32106, Thermo, Waltham, MA, USA), PrimeScript RT reagent Kit (Cat. RR047A, Takara Bio Inc., Shiga, Japan), TRIzol Reagent (Cat. 15596018, Thermo Fisher Scientific, Waltham, MA, USA), Universal SYBR Green Fast qPCR Mix (Cat. RM21203, ABclonal Technology Co., Ltd., Wuhan, China).

### 2.2. Neural induction of human ESC culture and VPA treatment

Human embryonic stem cell line H8 was obtained from Prof. Wei Jiang (Wuhan University). Cells were maintained by mTeSR1 medium with feeder cell free. Neural induction was conducted according to the protocol as reported with minor modification (Chambers et al., 2009). Briefly, human ESCs were digested using accutase and cultured in suspension using neural induction medium (NIM) for 7 days. After embryonic body (EB) formation, EBs were transferred to 6-well plates pre-coated with matrigel. Cells were cultured with NIM until rosette formation. Rosettes were digested by neural rosette selection reagent (Cat#05832, STEMCELL Technologies, Canada) and seeded onto coverslips pre-coated with matrigel and cultured with 50% neuron-differentiation medium (NDM), and 50% neurobasal containing 2% B27, 100 nmol/L brain-derived neurotrophic factor (BDNF, Cat. 45002, Peprotech) and 100 nmol/L glia-derived neurotrophic factor (GDNF, Cat. 45010, Peprotech) for 3 days. Then, cells were treated with VPA (1 mM, P4543, Sigma) or vehicle solution for 3 days as described (Meng et al., 2022).

For evaluating neuronal differentiation, cells were collected at 8 d after VPA treatment. For assessing metabolic changes, cells were analyzed at 3 d following VPA treatment. For interfering glycolysis, 2-DG was added at 3-5 d post VPA treatment.

### 2.3. Immunocytochemistry

For immunohistochemistry, cells were fixed with 4% paraformaldehyde phosphate buffer, blocked by PBS containing 3% BSA, and then incubated with primary antibodies overnight at room temperature as the following: rabbit anti-Sox2 (1:100, GTX101507, GeneTex), rabbit anti-Pax6 (1:100, GTX113241, GeneTex), rabbit anti-Ki67 (1:100, GTX16667, GeneTex), mouse anti-Tuj-1 (1:100, GT11710, GeneTex), mouse anti-CaMKII antibody (1:100, ab22609, Abcam), rabbit anti-VGLUT2 (1:100, 71555, Cell signaling technology), rabbit anti-Neurogenin 2 antibody (1:100, GTX129258, GeneTex); rabbit anti-Mash1 (1:100, Abcam, ab74065). After washing with PBS, corresponding secondary antibodies conjugated with donkey anti-rabbit (Alexa Fluor 594, 1:800, A-21207, Invitrogen), donkey anti-mouse (Alexa Fluor 594, 1:800, A-21203, Invitrogen), were incubated with the cells for 1 h at room temperature protected from light. After washing with PBS, sections were counterstained with DAPI (D9542, 1:1000, Sigma) for 20 min.

### 2.4. Western-blotting

Cells were homogenized in Radio Immunoprecipitation Assay (RIPA) lysis buffer containing proteinase inhibitors cocktail. Protein concentration was measured by bicinchoninic acid (BCA) assay. Protein samples were separated by 10–15% gel. After SDS-PAGE, protein was transferred to polyvinylidene fluoride (PVDF) membrane. Membranes were blocked with TBS containing 5% non-fat milk and 0.1% Tween 20 and then incubated with primary antibodies overnight at 4°C as the following: mouse anti-Tuj-1 (1:1000, GT11710, GeneTex), mouse anti-CaMKII antibody (1:1000, ab22609, Abcam), rabbit anti-VGLUT2 (1:1000, 71555, Cell signaling technology), rabbit anti-Pax6 (1:1000, GTX113241, GeneTex), rabbit anti-Histone-H3ac (1:3000, 616378, Active Motif), rabbit anti-Histone-H3 Polyclonal antibody (1:3000, 17168-1-AP, Proteintech), mouse anti-Histone-H3K9ac (1:3000, 91104, Active Motif), rabbit anti-Neurogenin 2 antibody (1:1000, GTX129258, GeneTex), rabbit anti-Mash1 (1:1000, ab74065, Abcam). After washing with TBST, membranes were incubated with HRP-conjugated anti-rabbit and HRP-conjugated anti-mouse (1:5000; Bioss, bs-0296G, and worldbio, WH-002) for 1 h at room temperature. Bands were visualized with an ECL kit (Cat. 32106, Thermo). Images were analyzed by ImageJ. For quantification of blots, the ratios of (gray scale of target protein)/(gray scale of β-actin) in experimental groups were compared to those of control groups.

### 2.5. Mitochondria staining

Cells were washed with DPBS. MitoSOX (10 μg/ml, Cat. M36008, Invitrogen) and TMRM (10 μg/ml, Cat. I34361, Invitrogen) were added directly into DPBS and incubated for 30 min. After 5 min’s washing with DPBS, images were taken under confocal microscope.

### 2.6. Mitochondrial respiratory complex activity, ATP levels, and extracellular acidification rate

The activity of mitochondrial respiratory complex I, II and IV, and ATP contents were measured by using commercial kits (BC0515, BC3235, BC0945, and BC0300, Solarbio, Beijing, China). The ECAR assay was conducted using a Seahorse XF-24 extracellular flux analyzer (Seahorse Bioscience, USA). Cells were seeded at a density of 8 × 10^5^ cells per well. One day before measurement, the sensor cartridge was calibrated overnight. ECAR were measured sequentially before or after the addition of following reagents: oligomycin (1 μM), glucose (10 mM) and 2-DG (50 mM). Each measurement cycle included the following steps: 2 min mixture, 1 min waiting, and 3 min measurement. Following the last assay cycle, the plate was removed and cell counting was conducted. Total cell data was used to normalize the measured ECAR values.

### 2.7. Realtime RT-PCR

Total RNA was extracted using TRIzol reagent. After reverse transcription, cDNA was mixed with PCR buffer and amplified using a standard protocol (95°C 1 min for initial degeneration, 95°C 5 s, 60°C 20 s cycles for amplification). The primers used were as the following:

*MCT-1*-F: GGAACATGGGCAAAGGAAGATTT

*MCT-1*-R: ATGTTCATGGCATCGGACTATTT

*LDH-A*-F: CCAGCGTAACGTGAACATCTTTAA

*LDH-A*-R: GACCCACCCATGACAGCTTAAT

*PKM2*-F: GACATTGATTCACCACCCATCAC

*PKM2*-R: GTTCAGACGAGCCACATTCATTC

*PDH*-F: ACGGTAGGGGAGCAGAGTG

*PDH*-R: ACACACACACATTCTCCGGG

*GAPDH*-F: AGAAGGCTGGGGCTCATTTG

*GAPDH*-R: AGGGGCCATCCACAGTCTTC.

### 2.8. Chromatin immunoprecipitation assay

Chromatin immunoprecipitation (ChIP) assay was conducted according to the manual of the ChIP assay kit (Upstate) as previously described (Wang et al., 2020). Briefly, human NPCs following VPA and 2-DG treatment were collected and fixed by 4% PFA for 15 min on ice. Sonication was performed to break chromatin. Rabbit anti-H3K9ac (1 mg/ml, AB10812, Abcam) was incubated with DNA–protein complex overnight at 4°C. Rabbit IgG at the same concentration was used as the negative control. The primer pairs targeting the core promoter regions of *Ngn2* and *Mash1* were as the following: (1) *Ngn2*-F: TAACTGGAGTGCCTGGGAGT, *Ngn2*-R: GTGCGTGTCTGTCTGTCAGT; (2) *Mash1*-F: GCGCAGCCTTAGTAGGAGAG, *Mash1*-R: GCAGAAGCAGCAGCAAAAGT.

### 2.9. TUNEL staining

TUNEL staining was performed as the manual of commercial kit (C1089, Beyotime, China). Briefly, cells were fixed by 4% PFA and washed by PBS. Then cells were incubated with 0.3% Triton X-100 in PBS and subsequently with TdT-mediated dUTP nick end labeling (TUNEL) staining solution for 60 min at 37°C.

### 2.10. Primary culture of mouse neural stem cells and treatment

Brains of E14–E17 mouse embryos were removed under a stereomicroscope. The cortex was dissected and digested in 0.125% trypsin for 10 min at 37°C. Cells were cultured in Neurobasal medium supplemented with 2% B27, 1% N2, 20 ng/ml epidermal growth factor (EGF), 20 ng/ml fibroblast growth factor (FGF) for 5–6 days. Primary neurospheres were digested and reseeded. VPA (1 mM) was added to secondary neurosphere for 3 days. Then neurospheres were digested and seeded on poly-lysine precoated coverslips, and cultured with 1% serum for 24 h.

### 2.11. Transmission electron microscope

Transmission electron microscope study was performed as described (Fan et al., 2016). Briefly, cells were fixed with a mixture of 4% paraformaldehyde and 0.05% glutaraldehyde. Then, cells were further fixed with 0.5% osmium tetroxide, dehydrated with graded ethanol, replaced with propylene oxide, and directly flat-embedded in Epon 812. Ultrathin sections (70–80 nm) were prepared on an LKB Nova Ultratome. After being counterstained with uranyl acetate and lead citrate, the sections were examined under an electron microscope (JEM-1230).

### 2.12. Data analysis

For immunocytochemistry and Western-blots, at least 3 biological repeats were performed for each experiment. For taking images of immunocytochemistry, at least five images were taken randomly from each cover-slip by a researcher blind to the experimental design. The data were presented as means ± standard error of mean (S.E.M.). Data were analyzed by one-way analysis of variance (ANOVA) or two-tailed Student’s *t*-test using SPSS l6.0 (Chicago, IL, USA). *P*-values less than 0.05 were considered as statistically significant.

## 3. Results

### 3.1. VPA treatment stimulates excitatory neuron differentiation of human NPC

**FIGURE 1:**
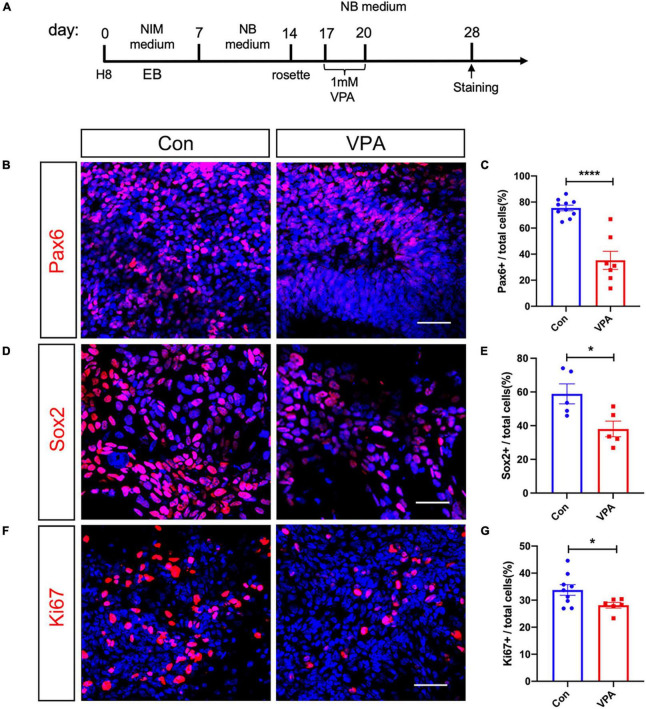
Effects of VPA-treatment on the expression of neural progenitor and cell cycle markers of human NPCs. **(A)** Experimental design. **(B,C)** Immunostaining and quantification of Pax6 in control and VPA-treated human NPCs. **(D,E)** Immunostaining and quantification of Sox2 in control and VPA-treated human NPCs. **(F,G)** Immunostaining and quantification of Ki67 in control and VPA-treated human NPCs. VPA treatment significantly reduced the expression of Pax6, Sox2, and Ki67. Bars = 50 μm. *N* = 3 batches of cells. Student’s *t*-test. *P < 0.05. ****P < 0.0001. NIM, neural induction medium. EB, embryonic body. NB, neurobasal. VPA, valproic acid. Con, control.

We adopted a standard neural induction protocol to induce neurons from human embryonic stem cell (ESC) line H8. Following rosette passaging, approximately 80% of cells expressed neural progenitor markers Pax6 and Nestin (Supplementary Figures 1A, B). After 3 weeks’ culture in neural differentiation medium, 88.36 ± 2.29% of cells expressed neuronal marker Tuj-1 (Supplementary Figures 1C, D), suggesting the success of neural induction. VPA (1 mM) was added for 3 d after rosette appearance (Figure 1A). Eight days after VPA treatment, we examined the proliferation of human NPCs and the expression of neural progenitor markers. The percentages of Pax6-positive cells dropped from 75.45 ± 1.99% to 35.26 ± 0.85% in VPA-treated cells (Figures 1B, C). Similarly, the percentages of Sox2-positive cells decreased by approximately 22.8% in VPA treated cells (Figures 1D, E). Ki67 staining showed that VPA treatment significantly reduced the cells undergoing proliferation (Figures 1F, G). These data indicated that VPA suppressed the proliferation and expression of neural progenitor markers of human NPCs.

**FIGURE 2:**
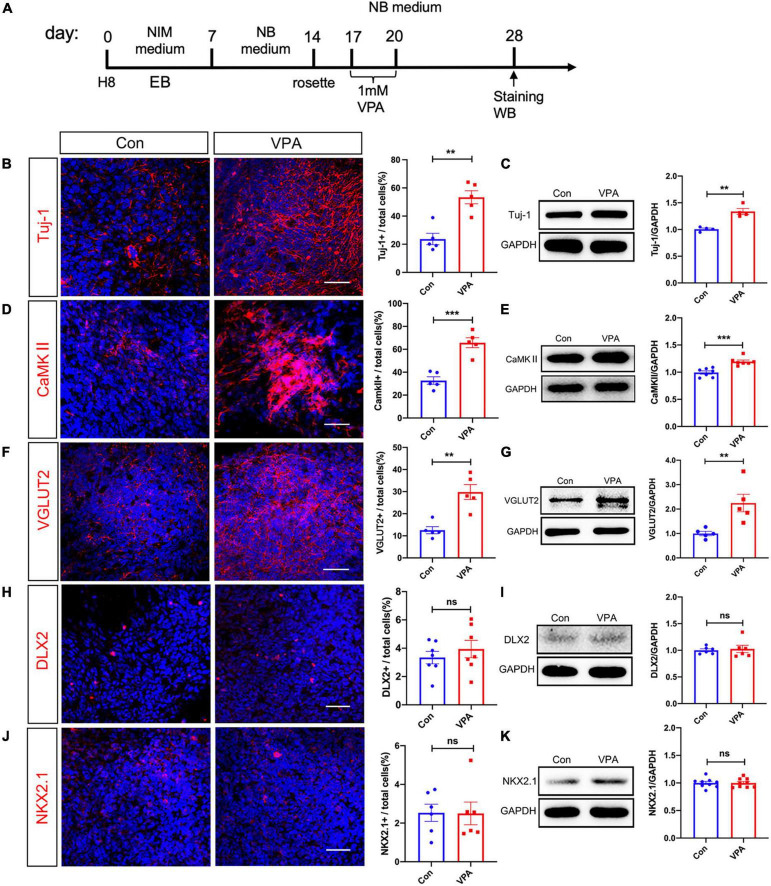
Effects of VPA-treatment on the expression of excitatory and inhibitory neuronal markers of human NPCs. **(A)** Experimental design. **(B,C)** Immunostaining and Western-blotting of Tuj-1 in control (Con) and VPA-treated human NPCs. **(D,E)** Immunostaining and Western-blotting of CaMKII in control (Con) and VPA-treated human NPCs. **(F,G)** Immunostaining and Western-blotting of VGLUT2 in control (Con) and VPA-treated human NPCs. Notice the increase of Tuj-1, CaMKII and VGLUT2 in VPA-treated cells. **(H,I)** Immunostaining and Western-blotting of DLX2 in control (Con) and VPA-treated human NPCs. **(J,K)** Immunostaining and Western-blotting of NKX2.1 in control (Con) and VPA-treated human NPCs. Bars = 50 μm. *N* = 3 batches of cells. Student’s *t*-test. **P < 0.01. ***P < 0.001. ns means no significance. NIM, neural induction medium. EB, embryonic body. NB, neurobasal. VPA, valproic acid. Con, control.

We next examined the expression of neuron-specific markers at the same time point after VPA treatment (Figure 2A). Immunostaining of Tuj-1, a pan-neuronal marker, showed a 70% increase of Tuj-1-positive cells in VPA-treated cells (Figure 2B). Western-blotting confirmed the up-regulation of Tuj-1 by VPA treatment (Figure 2C). To explore if VPA treatment influenced the differentiation of hNPCs toward excitatory or inhibitory neurons, we examined the expression of excitatory neuronal markers (CaMKII and VGLUT2) and markers of inhibitory neuronal progenitors (DLX2 and NKX2.1). VPA treatment stimulated the percentages of CaMKII-positive cells from 32.74 ± 3.25% to 65.71 ± 4.38% (Figure 2D), and the percentages of VGLUT2-positive cells from 12.54 ± 1.60% to 29.80 ± 3.37% (Figure 2F). The increase of CaMKII and VGLUT2 was confirmed by Western-blotting (Figures 2E, G). In terms of inhibitory neuronal markers, both immunocytochemistry and Western-blotting showed no change of DLX2 and NKX2.1 expression following VPA treatment (Figures 2H–K). These data demonstrated that VPA treatment biased the differentiation of human NPCs toward excitatory neurons.

Previous study reported that VPA treatment could promote the differentiation of projection neurons in mouse brain (Fujimura et al., 2016). To test if VPA treatment also affected excitatory neuron differentiation in mouse, we treated primary neural stem cells from C57 mouse with same procedure as human NPCs. After 24 h’s differentiation, immunostaining showed significant increase of both Tuj-1-positive and CaMKII-positive cells in VPA treated cells, as compared with vehicle control (Supplementary Figure 2).

### 3.2. VPA impairs mitochondrial function and increases glycolysis of human NPC

**FIGURE 3:**
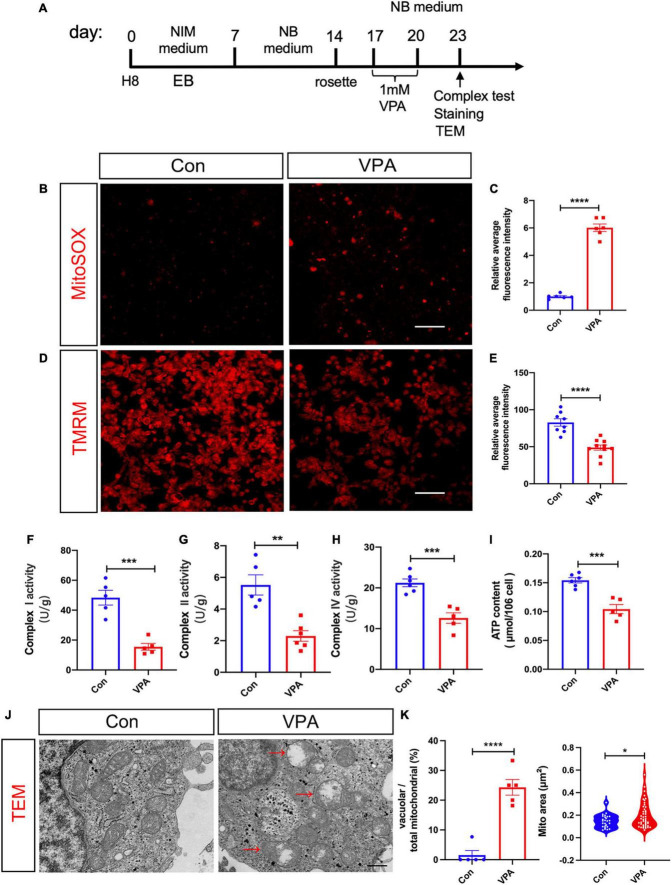
Effects of VPA-treatment on the mitochondrial function of human NPCs. **(A)** Experimental design. **(B,C)** Representative images of MitoSOX staining and its quantification on human NPCs treated with or without VPA. **(D,E)** Representative images of TMRM staining and its quantification on human NPCs treated with or without VPA. VPA-treatment significantly increased the fluorescent intensity of MitoSOX but decreased that of TMRM. **(F–H)** Mitochondrial respiratory complex activity of human NPCs treated with or without VPA. **(I)** ATP levels of human NPCs treated with or without VPA. Notice the decrease of ATP levels and activity of mitochondrial complex I, II, and IV. **(J,K)** Representative transmission electron microscopic images of cells treated with or without VPA, and the quantification. Notice the vacuolar mitochondria in VPA-treated cells (arrows). Bars = 50 μm in panels **(B,D)** and 500 nm in panel **(J)**. *N* = 3 batches of cells. Student’s *t*-test. *P < 0.05. **P < 0.01. ***P < 0.001. ****P < 0.0001. NIM, neural induction medium. EB, embryonic body. NB, neurobasal. TEM, transmission electron microscope. VPA, valproic acid. Con, control.

To test if VPA treatment affect mitochondrial function, we performed MitoSOX (an indicator of mitochondrial superoxide) and tetramethylrhodamine methyl ester (TMRM, an indicator of mitochondrial membrane potential) staining at 3 days post VPA treatment (Figure 3A). In comparison with vehicle control, VPA-treated human NPCs exhibited much stronger MitoSOX fluorescent intensity and much weaker TMRM intensity (Figures 3B–E). To further evaluate mitochondrial function, we measured the respiratory complex activity and ATP content in VPA-treated cells versus control cells. The results showed significant lower activities of respiratory complex I, II, and IV and reduced levels of ATP (Figures 3F–I). These data indicated that VPA treatment may impair the oxidative phosphorylation of mitochondria, thus leading to less ATP production. Further, we assessed the effects of VPA treatment on the morphology of mitochondria. Electron microscope revealed remarkable vacuolar mitochondria in the cytoplasm of VPA-treated cells. In addition, the average area of mitochondria was significantly increased in VPA-treated cells (Figures 3J, K).

**FIGURE 4:**
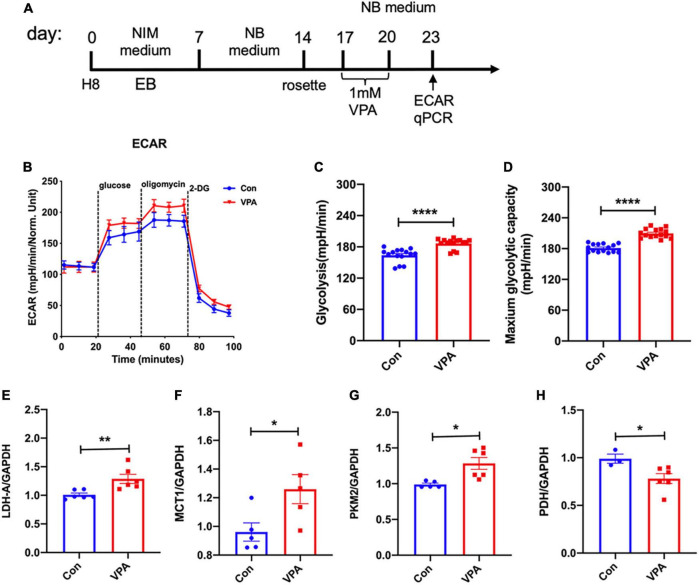
Effects of VPA-treatment on the glycolytic activity of human NPCs. **(A)** Experimental design. **(B–D)** ECAR assay of human NPCs treated with or without VPA. Notice the increase of glycolytic activity and maximum glycolytic capacity by VPA treatment. **(E–H)** Realtime RT-PCR of LDH-A, MCT1, PKM2, and PDH in human NPCs treated with or without VPA. *N* = 3 batches of cells. Student’s *t*-test. *P < 0.05. **P < 0.01. ****P < 0.0001. ECAR, extracellular acid rate. NIM, neural induction medium. EB, embryonic body. NB, neurobasal. VPA, valproic acid. Con, control.

Considering that glycolysis is an alternative pathway for ATP production, we evaluated glycolytic activity by extracellular acidification rate (ECAR, Figure 4A). ECAR assay showed that VPA-treated cells exhibited significantly higher levels of basal glycolytic activity upon glucose stimulation, and higher levels of maximal glycolytic activity upon oligomycin stimulation (Figures 4B–D). To confirm the change of glycolysis, we assessed the expression of key enzymes in glycolytic pathway. Realtime RT-PCR showed significantly increased mRNA levels of lactate dehydrogenase A (LHD-A), monocarboxylate transporter 1 (MCT-1), pyruvate kinase 2 (PKM2) and significantly decreased mRNA levels of pyruvate dehydrogenase (PDH) (Figures 4E–H). These data supported the elevation of glycolysis by VPA-treatment.

### 3.3. Inhibiting glycolysis rescues VPA-stimulated excitatory neuron differentiation

**FIGURE 5:**
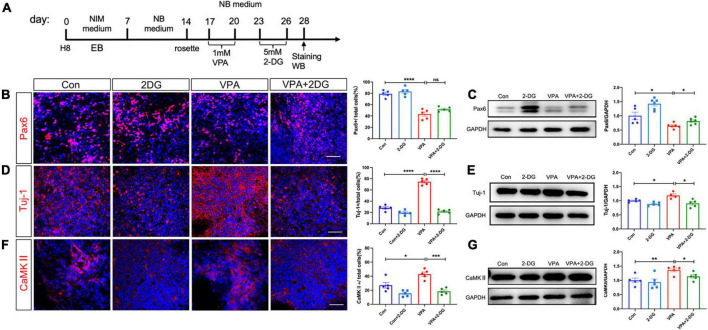
Effects of 2-DG treatment on VPA-induced excitatory neuron differentiation of human NPCs. **(A)** Experimental design. **(B,C)** Immunocytochemistry and Western-blotting of Pax6 in control cells, cells treated with 2-DG, cells treated with VPA, and cells treated with both VPA and 2-DG. **(D,E)** Immunocytochemistry and Western-blotting of Tuj-1 in control cells, cells treated with 2-DG, cells treated with VPA, and cells treated with both VPA and 2-DG. **(F,G)** Immunocytochemistry and Western-blotting of CaMKII in control cells, cells treated with 2-DG, cells treated with VPA, and cells treated with both VPA and 2-DG. Notice the rescue effects of 2-DG on the expression of Pax6, Tuj-1 and CaMKII. Bars = 50 μm. *N* = 3 batches of cells. ANOVA. *P < 0.05. **P < 0.01. ***P < 0.001. ****P < 0.0001. ns means no significance. NIM, neural induction medium. EB, embryonic body. NB, neurobasal. VPA, valproic acid. Con, control.

Recent studies reported that NPC-to-neuron differentiation is accompanied by a glycolysis-to-oxidative phosphorylation switch (Zheng et al., 2016). We then asked if this elevated glycolysis were involved in the VPA induced differentiation of excitatory neurons. 2-deoxy-d-glucose (2-DG), a mimic of glucose which can inhibit glycolysis, was added into culture medium for 3 days following VPA-treatment (Figure 5A). 2-DG remarkably up-regulated the numbers of Pax6-positive cells and Sox2-positive cells in VPA-treated cells (Figure 5B; Supplementary Figure 3A). As to neuronal markers, 2-DG significantly reduced the percentage of cells expressing Tuj-1, CaMKII, and VGLUT2 in VPA-treated cells to a level similar as normal control (Figures 5D, F; Supplementary Figure 3B). Western-blotting confirmed the restoration of the expression levels of Pax6, Tuj-1, and CaMKII by 2-DG in VPA-treated cells (Figures 5C, E, G). These data demonstrated an involvement of glycolysis in the VPA-induced excitatory neuron differentiation.

To evaluate if VPA and 2-DG influenced the survival of human NPCs, we performed TUNEL staining. No difference of TUNEL-positive cells was found among control cells, VPA-treated cells and 2-DG treated cells (Supplementary Figure 4).

### 3.4. Histone acetylation of Ngn2 and Mash1 is regulated by glycolysis

**FIGURE 6:**
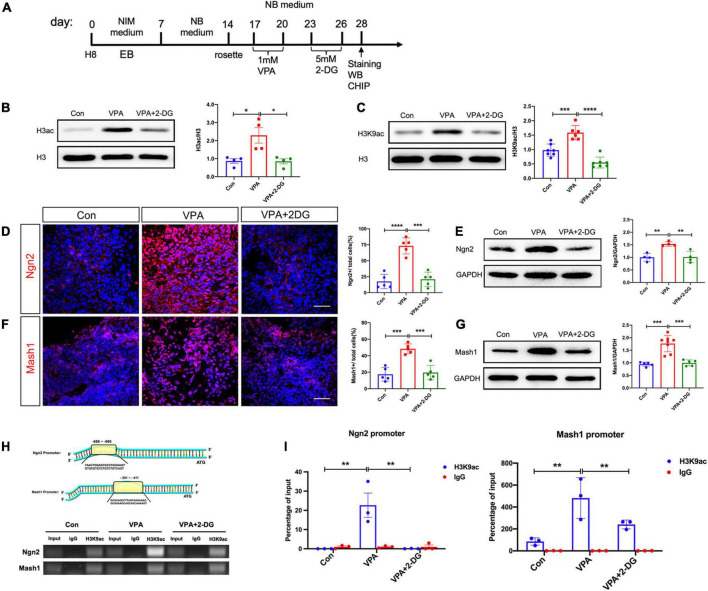
Effects of VPA and 2-DG treatment on the histone acetylation of Ngn2 and Mash1. **(A)** Experimental design. **(B,C)** Western-blotting of H3ac and H3K9ac in control cells, cells treated with VPA, and cells treated with both VPA and 2-DG. 2-DG treatment effectively blocked the VPA-induced up-regulation of H3ac and H3K9ac. **(D,E)** Immunocytochemistry and Western-blotting of Ngn2 in control cells, cells treated with VPA, and cells treated with both VPA and 2-DG. **(F,G)** Immunocytochemistry and Western-blotting of Mash1 in control cells, cells treated with VPA, and cells treated with both VPA and 2-DG. **(H,I)** ChIP assay of the binding of H3K9ac on the core promotor region of Ngn2 and Mash1. Bars = 50 μm. *N* = 3 batches of cells. ANOVA. *P < 0.05. **P < 0.01. ***P < 0.001. ****P < 0.0001. NIM, neural induction medium. EB, embryonic body. NB, neurobasal. VPA, valproic acid. Con, control.

Previous studies have reported that VPA induces neural differentiation mainly *via* modulating histone acetylation (Yu et al., 2009; Zhang et al., 2016). We thus examined if glycolysis were involved in VPA-induced histone acetylation (Figure 6A). Western-blotting showed that VPA treatment significantly increased the protein levels of H3ac and H3K9ac (Figures 6B, C). The increase of H3ac and H3K9ac was significantly compromised by 2-DG treatment (Figures 6B, C). Next, we explored if glycolysis were involved in the expression of neuron specific transcription factors. Both immunocytochemistry and Western-blotting showed that 2-DG treatment effectively blocked the induction of Ngn2 and Mash1, two excitatory neuronal fate determinants, in VPA-pretreated cells (Figures 6D–G). Further, we explored the binding of H3K9ac on the promoter region of Ngn2 and Mash1 in control cells, VPA-treated cells, and cells treated with VPA and 2-DG. Chromatic co-immunoprecipitation assay showed that VPA induced strong binding of H3K9ac on the promoter of Ngn2 and Mash1, while 2-DG treatment remarkably compromised the binding of H3K9ac on the promoter of Ngn2 and Mash1 (Figures 6H–I). These data indicated that glycolysis might actively contribute to the histone acetylation process during excitatory neuron differentiation.

## 4. Discussion

In the present study, we investigated the involvement of glycolysis in VPA-induced excitatory neuron differentiation by using a well-established human embryonic cell line. By checking the expression of neuronal markers, we observed an arrest of human NPC proliferation and increase of excitatory neurons production by VPA treatment. By examining the mitochondrial respiratory complex activity, extracellular acidification rate, expression of glycolytic enzymes and ultrastructural morphology, we detected an impairment of mitochondrial function and elevation of glycolysis shortly after VPA stimulation. Further, our data showed that suppressing glycolysis not only rescued differentiation of human NPCs, but also attenuated the binding of H3K9ac on the promoter region of Ngn2 and Mash1. Taken together, these data disclosed an unexpected role of glycolysis in the VPA-induced excitatory neuron differentiation through modulating the histone acetylation of neuron specific transcription factors.

In animal experiments, most studies focused on the synaptic toxicity of VPA which affected both the pre- and post-synaptic responses and differently influenced the development of excitatory and inhibitory synapses (Cunningham et al., 2003; Wang et al., 2012; Traetta et al., 2021). At the circuit level, aberrant development of striatal compartments and corticostriatal pathway were found in VPA-induced ASD mice (Kuo and Liu, 2017). These reports supported the increase of synaptic E-I ratio (Qi et al., 2022). At the neuronal differentiation level, previous studies have documented early neuron-inducing effects of VPA, but not analyzing the specific neuronal sub-type differentiation. By comparing the expression of excitatory neuronal markers (CaMKII and VGLUT2) and inhibitory neuronal progenitor markers (DLX2 and NKX2.1), we demonstrated a specific effect of VPA on excitatory neuronal differentiation. This may add a new dimension on the explanation of increased “E-I” ratio of VPA-induced ASD mice.

As a histone deacetylase (HDAC) inhibitor, the effect of VPA on neural development is usually attributed to histone acetylation. Our observation of up-regulating H3ac and H3K9ac is in line with previous studies (Hezroni et al., 2011; Boudadi et al., 2013). Interestingly, our data showed a rapid impairment of mitochondrial function and elevation of glycolysis in human NPCs following VPA treatment. One study has reported ultrastructural disorganization of mitochondria in human NPCs following VPA treatment (Costa et al., 2018). Our data not only confirmed the morphological change of mitochondria, but also demonstrated an impairment of respiratory complex activity and increase of glycolysis in human NPCs by VPA. So far as we know, two papers reported different effects of VPA on glycolysis (Fang et al., 2019; Salsaa et al., 2020). Our data of ECAR assay and expression of glycolytic enzymes supported the elevation of glycolysis by VPA. Suppressing glycolysis *via* 2-DG abolished the VPA-induced excitatory neuron differentiation. In our parallel study, we observed that VPA treatment activated Wnt signaling in human NPCs (data not shown). In tumor cells, Wnt signaling up-regulate glycolysis by directly targeting glycolytic enzymes such as pyruvate dehydrogenase kinase, isozyme 1 (PDK1) (Zuo et al., 2021). All these support our observation that VPA treatment up-regulate glycolysis in human NPCs.

Recently, it is becoming recognized that cellular metabolites or metabolic pathways play important roles in modulating epigenetic modification, thus affecting stem cell identity maintenance and cell fate specification (Ryall et al., 2015). The products of tricarboxylic acid cycle actively regulate histone demethylation (Okabe et al., 2020). The metabolites of one-carbon DNA are involved in DNA methylation (Mentch and Locasale, 2016). Relatively, how glycolysis crosstalk with epigenetic modifications has been less studied. The most important mechanistic finding of present study, in our eyes, is the attenuation of H3K9 acetylation at the promoter of Ngn2 and Mash1 by 2-DG treatment. This disclosed a new dimension of glycolysis in epigenetic modification. In pluripotent stem cells, glycolysis produces acetyl-CoA, which in turns regulate histone deacetylation (Moussaieff et al., 2015). Lactate, the major product of glycolysis, can regulate gene expression *via* inducing histone lactylation (Zhang et al., 2019). In our experimental setting, whether glycolysis modulate histone acetylation *via* acetyl-CoA or *via* competitive effects of histone lactylation, merits detailed investigation in the future.

## Data availability statement

The original contributions presented in this study are included in the article/Supplementary material, further inquiries can be directed to the corresponding authors.

## Author contributions

AC, MW, and CX performed most of the experiments and collected and analyzed the data. YZ and WZ contributed to the human ES cell culture. PX contributed to the ECAR assay. YL and XY contributed to the immunocytochemistry. YW and SW conceived and supervised the project, provided financial support, and wrote the manuscript. All authors contributed to the article and approved the submitted version.

## Supplementary material

Supplementary Figure 1 **(A,B)** Experimental design, immunocytochemistry and quantification of Pax6 and Nestin in human ESC and NPCs. **(C,D)** Experimental design, immunocytochemistry and quantification of Tuj-1 in human ESC and human ESC derived neurons. Bars = 50 μm. N = 3 batches of cells. Student’t test. *****P* < 0.0001.

Supplementary Figure 2 Effects of VPA on the differentiation of mouse neural stem cells. **(A)** immunostaining of Tuj-1 in control and VPA-treated mouse neural stem cells. **(B)** immunostaining of CaMKII in control and VPA-treated mouse neural stem cells. Bars = 50 μm. N = 3 batches of cells. Student’t test. ****P* < 0.001.

Supplementary Figure 3 Effects of 2-DG treatment on the VPA-induced expression of Sox2 and VGLUT2. **(A)** Immunocytochemistry and quantification of Sox2 in normal cultured human NPCs (Con), 2-DG treated, VPA treated, VPA plus 2-DG treated human NPCs. **(B)** Immunocytochemistry and quantification of VGLUT2 in normal cultured human NPCs (Con), 2-DG treated, VPA treated, VPA plus 2-DG treated human NPCs. Bars = 50 μm. N = 3 batches of cells. ANOVA. **P* < 0.05. ***P* < 0.01. *****P* < 0.0001.

Supplementary Figure 4 Effects of 2-DG and VPA on cell death. TUNEL staining showed no significant difference of cell death among cells treated with or without 2-DG or VPA. Bar = 50 μm. N = 3 batches of cells. ANOVA.
