# Supplementary figures and images for: Glycolysis mediates neuron specific histone acetylation in valproic acid-induced human excitatory neuron differentiation
Source: Front Mol Neurosci. 2023 Apr 6;16:1151162. doi: 10.3389/fnmol.2023.1151162 (PMC10118002; doi:10.3389/fnmol.2023.1151162)

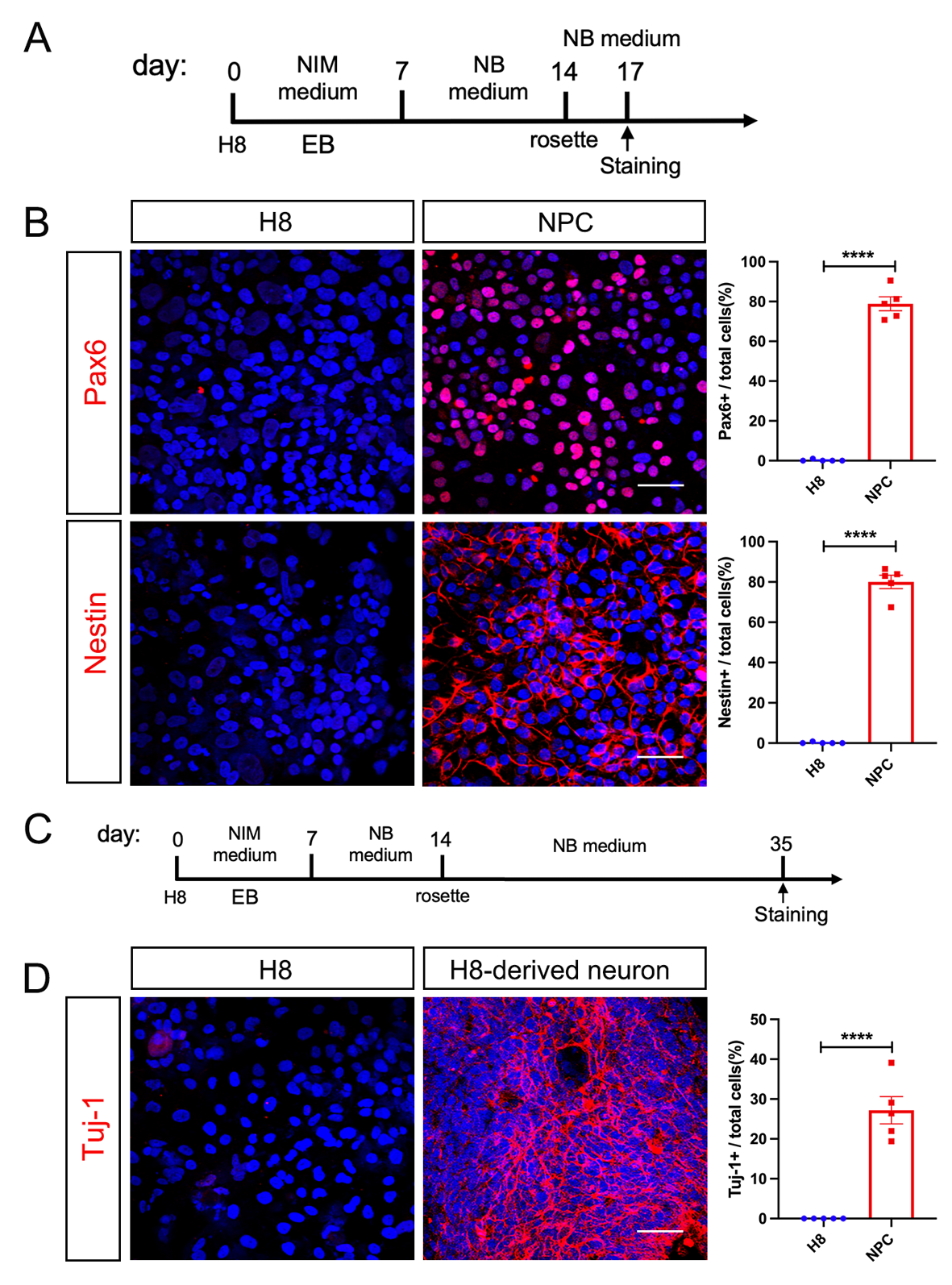

Supplement: Supplementary Figure 1 — (A,B) Experimental design, immunocytochemistry and quantification of Pax6 and Nestin in human ESC and NPCs. (C,D) Experimental design, immunocytochemistry and quantification of Tuj-1 in human ESC and human ESC derived neurons. Bars = 50 μm. N = 3 batches of cells. Student’t test. ****P < 0.0001. [file Image_1.tif]

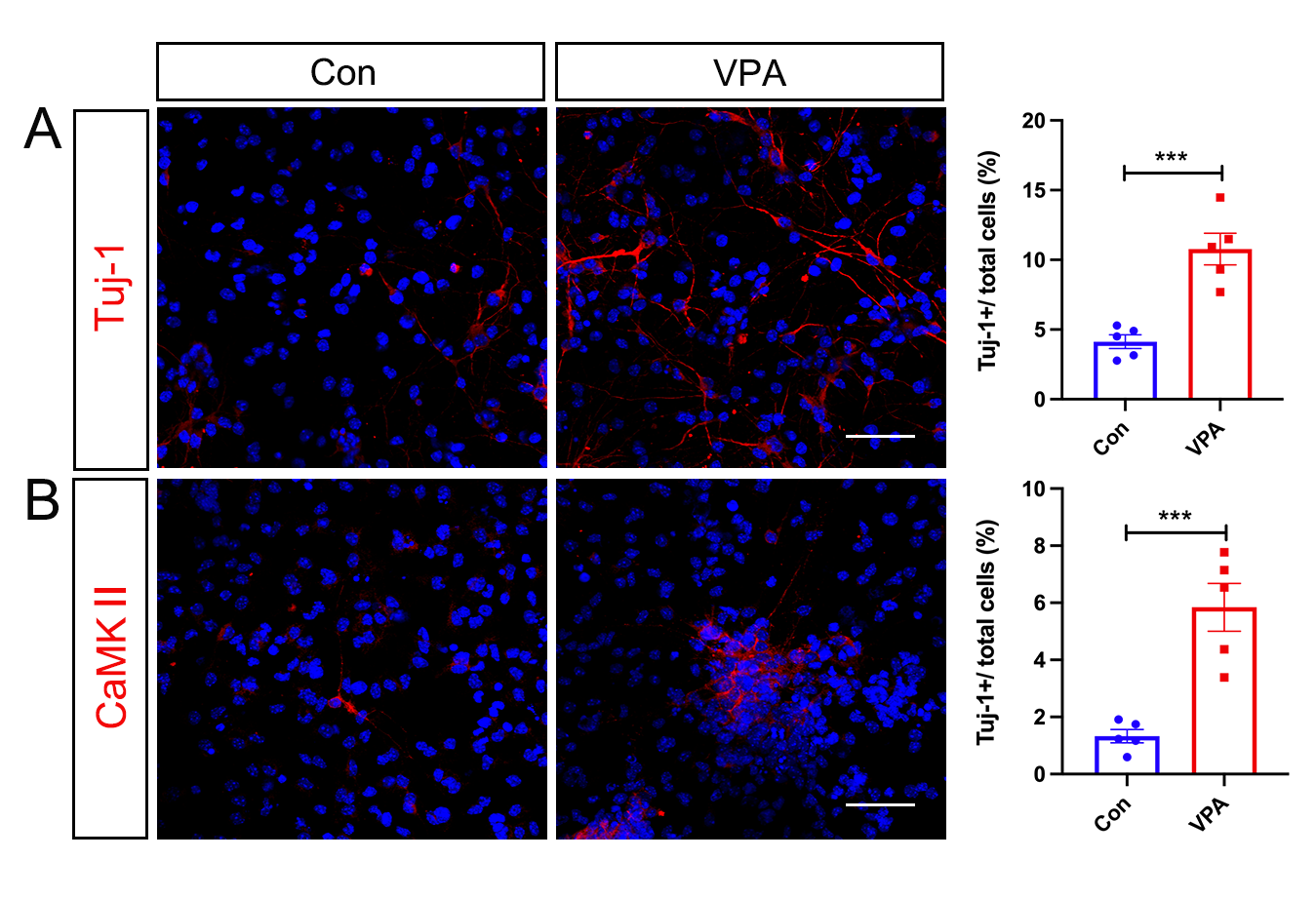

Supplement: Supplementary Figure 2 — Effects of VPA on the differentiation of mouse neural stem cells. (A) immunostaining of Tuj-1 in control and VPA-treated mouse neural stem cells. (B) immunostaining of CaMKII in control and VPA-treated mouse neural stem cells. Bars = 50 μm. N = 3 batches of cells. Student’t test. ***P < 0.001. [file Image_2.TIF]

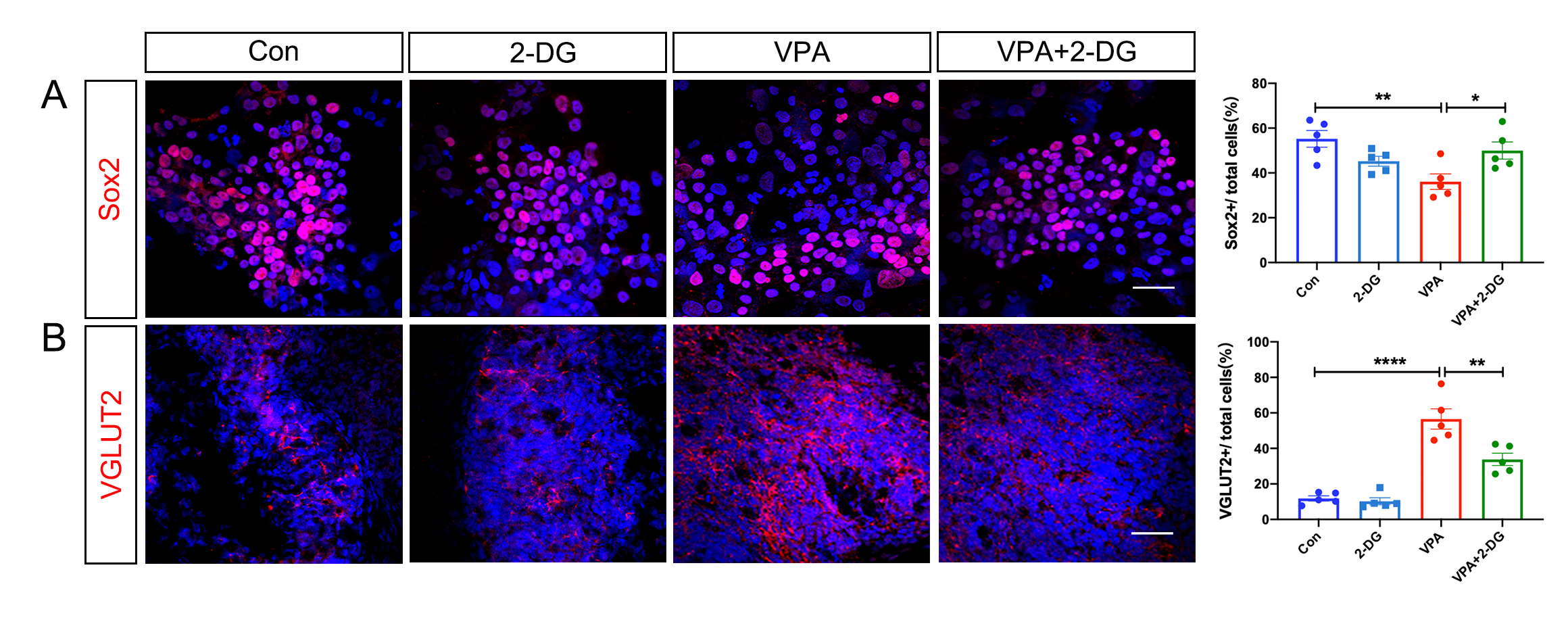

Supplement: Supplementary Figure 3 — Effects of 2-DG treatment on the VPA-induced expression of Sox2 and VGLUT2. (A) Immunocytochemistry and quantification of Sox2 in normal cultured human NPCs (Con), 2-DG treated, VPA treated, VPA plus 2-DG treated human NPCs. (B) Immunocytochemistry and quantification of VGLUT2 in normal cultured human NPCs (Con), 2-DG treated, VPA treated, VPA plus 2-DG treated human NPCs. Bars = 50 μm. N = 3 batches of cells. ANOVA. *P < 0.05. **P < 0.01. ****P < 0.0001. [file Image_3.TIF]

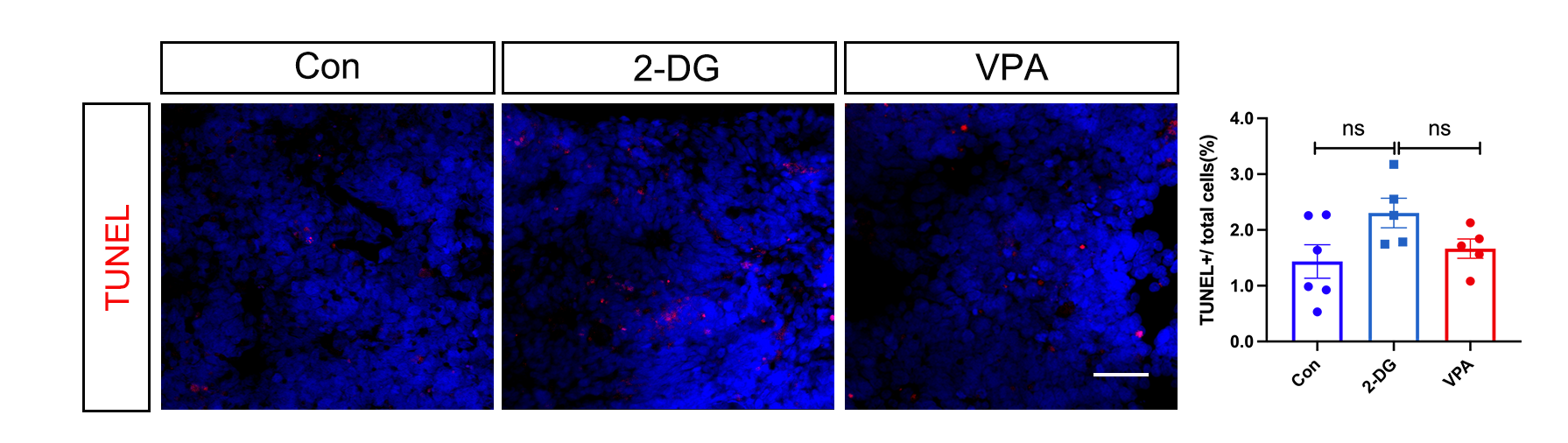

Supplement: Supplementary Figure 4 — Effects of 2-DG and VPA on cell death. TUNEL staining showed no significant difference of cell death among cells treated with or without 2-DG or VPA. Bar = 50 μm. N = 3 batches of cells. ANOVA. [file Image_4.TIF]

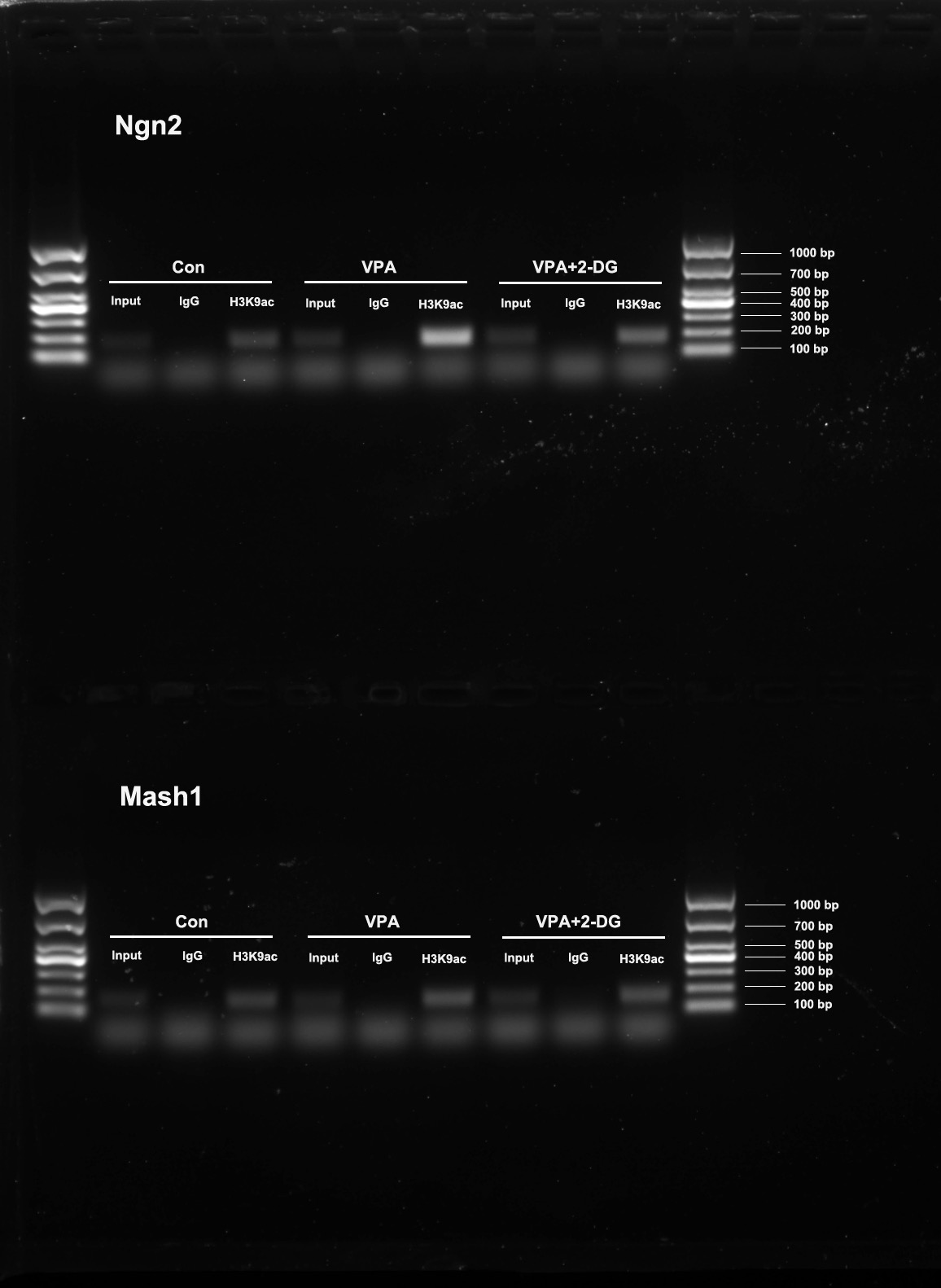

Supplement: Supplementary file 5 [file Image_5.JPEG]

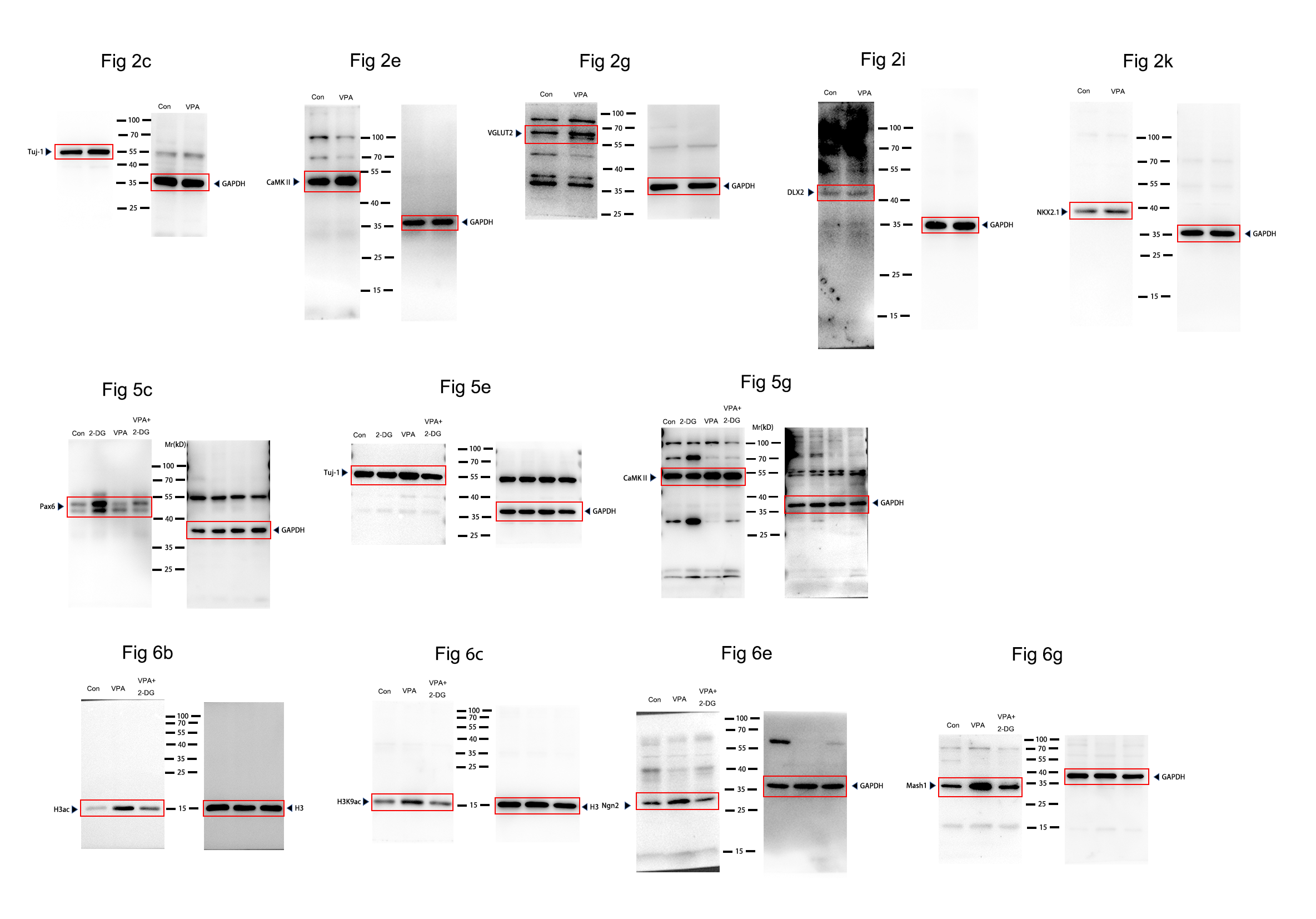

Supplement: Supplementary file 6 [file Image_6.TIF]
